# Visual Field Pointwise Analysis of the Idiopathic Intracranial Hypertension Weight Trial (IIH:WT)

**DOI:** 10.1167/tvst.12.5.1

**Published:** 2023-05-01

**Authors:** Susan P. Mollan, Samuel Bodoza, Áine Ní Mhéalóid, James L. Mitchell, Neil R. Miller, Giovanni Montesano, David P. Crabb, Michael Wall, Kristian Brock, Alexandra J. Sinclair

**Affiliations:** 1 Birmingham Neuro-Ophthalmology, Queen Elizabeth Hospital Birmingham, University Hospitals Birmingham NHS Foundation Trust, Birmingham, UK; 2 Informatics, Queen Elizabeth Hospital Birmingham, University Hospitals Birmingham NHS Foundation Trust, Birmingham, UK; 3 Division of Optometry and Visual Sciences, School of Health Sciences, City University of London, London, UK; 4 Wilmer Eye Institute, The Johns Hopkins University School of Medicine, Baltimore, MD, USA; 5 NIHR Biomedical Research Centre, Moorfields Eye Hospital NHS Foundation Trust, UCL Institute of Ophthalmology, London, UK; 6 Department of Neurology, Queen Elizabeth Hospital, University Hospitals Birmingham NHS Foundation Trust, Birmingham, UK; 7 Cancer Research Clinical Trials Unit, College of Medical and Dental Sciences, University of Birmingham, Birmingham, UK; 8 Institute of Metabolism and Systems Research, College of Medical and Dental Sciences, University of Birmingham, Birmingham, UK

**Keywords:** visual field, point sensitivity, idiopathic intracranial hypertension, pseudotumor cerebri, perimetric mean deviation

## Abstract

**Purpose:**

This study was designed to determine if point analysis of the Humphrey visual field (HVF) is an effective outcome measure for people with idiopathic intracranial hypertension (IIH) compared with mean deviation (MD).

**Methods:**

Using the IIH Weight Trial data, we performed a pointwise analysis of the numerical retinal sensitivity. We then defined a medically treated cohort as having MDs between −2 dB and −7 dB and calculated the number of points that would have the ability to change by 7 dB.

**Results:**

The HVF 24-2 mean ± SD MD in the worse eye was −3.5 ± 1.1 dB (range, −2.0 to −6.4 dB). Total deviation demonstrated a preference for the peripheral and blind spot locations to be affected. Points between 0 dB and −10 dB demonstrated negligible ability to improve, compared with those between −10 dB and −25 dB. For the evaluation of the feasibility for a potential medical intervention trial, only 346 points were available for analysis between −10 dB and −25 dB bilaterally, compared with 4123 points in baseline sensitivities of 0 to −10 dB.

**Conclusions:**

Patients with IIH have mildly affected baseline sensitivities in the visual field based on HVF analyzer findings, and the majority of points do not show substantial change over 24 months in the setting of a randomized clinical trial. Most patients with IIH who are eligible for a medical treatment trial generally have the mildest affected baseline sensitivities. In such patients, pointwise analysis offers no advantage over MD in detection of visual field change.

## Introduction

Idiopathic intracranial hypertension (IIH) is characterized by raised intracranial pressure (ICP) associated, in most cases, with papilledema, visual field defects, and, in some cases, permanent visual loss.^1^ Most people with IIH have moderate to severe headaches, systemic metabolic dysfunction, and central obesity.^2^^–^^4^ The incidence of IIH is increasing around the world, commensurate with the increase in worldwide obesity.^5^^,^^6^ Depending on the severity of IIH, patients can be treated with weight loss alone, ICP-lowering medications such as acetazolamide, ICP-lowering surgery, or a combination of these. The IIH Weight Trial (IIH:WT) showed that weight loss achieved by bariatric surgery resulted in long-term remission of ICP compared with a lifestyle weight-management intervention.^7^

The Humphrey visual field (HVF) mean deviation (MD) has been used as an endpoint in IIH clinical trials.^8^^,^^9^ However, although the IIH:WT met its primary endpoint (change in ICP measured by lumbar puncture opening pressure at 12 months), there was no significant improvement seen in the MD in either arm. We thus wondered if a different method—pointwise analysis—might be a more sensitive indicator of a change in the visual field in IIH patients participating in a treatment trial.

There are a number of different ways to evaluate visual field damage.^10^^–^^13^ The MD determined by the HVF Analyzer (Carl Zeiss Meditec, Dublin, CA) is measured in decibels (dB) using a logarithmic scale and determines the average difference in visual field sensitivity compared with the mean sensitivity of a normal person of the same age. Weighting is inversely proportional to the expected variance at each location in a normal population, effectively giving more weight to the central locations.^14^^–^^16^ A key regulator, the US Food and Drug Administration, considers a change of 7 dB in MD to be acceptable as being clinically meaningful.^17^ In IIH, the expected MD change is smaller compared with other optic neuropathies such as glaucoma. For most patients in an IIH medical intervention trial, a 7-dB change would be unachievable, as the MD inclusion criteria would likely be between −2 dB and −7 dB, which would represent a floor effect.

Another functional endpoint that has been recommended for an optic neuropathy treatment trial is a change of 7 dB in five or more predefined reproducible visual locations.^17^ Restricting an analysis to a particular subset of points in the visual field has not been previously prospectively investigated in IIH; however, the IIH Treatment Trial (IIHTT) investigators performed a post hoc pointwise analysis of the HVF. For each of the 52 points, a linear regression analysis was performed with the decibel measurement as the outcome variable and time as the independent variable. The IIHTT investigators demonstrated that peripheral points were more affected than central points. Although the magnitude of change in points was modest, there was significantly more improvement in the acetazolamide treatment arm.^10^ Given the lack of correlation in the IIH:WT outcome measures and MD, we hypothesized that a pointwise analysis of the IIH:WT visual field data could potentially reveal localized improvements not demonstrated by the MD. The number of participants required and the number of points that could be predicted to change in an IIH trial population could be determined. The purpose of this study was to assess if point analysis of the HVF would be feasible in a cohort of people with active IIH in the setting of a randomized clinical trial.

## Materials and Methods

IIH:WT was a prospective, multi-center, open-label, parallel-group, controlled trial in which participants with IIH were randomized in a 1:1 ratio to a bariatric surgery pathway or the Weight Watchers program, a community weight management intervention (CWI). The study was approved by the Ethics Review Board of the National Research Ethics Committee West Midlands, and the Black Country approved IIH:WT (14/WM/0011). In accordance with the Declaration of Helsinki, all subjects gave written informed consent to participate in the study, and the detailed clinical trial methodology has been published.^18^ Anonymized individual participant data will be made available along with the trial protocol and statistical analysis plan. Proposals should be made to the corresponding author and will be reviewed by the Birmingham Clinical Trials Unit Data Sharing Committee in discussion with the chief investigator. A formal data sharing agreement may be required after release of the data has been approved and before the data can be released.

### Subjects

Women (18–55 years old) with a body mass index (BMI) > 35 kg/m2 were eligible if they had a clinical diagnosis of active IIH according to criteria outlined by Friedman et al.^19^ All participants were recruited between March 2014 and October 2017. Evaluations were performed at baseline, 12 months, and 24 months.^18^ The primary outcome was ICP as measured by lumbar puncture; secondary outcomes have been reported elsewhere.^7^^,^^18^ At each visit, HVF with a 24-2 Swedish interactive threshold algorithm standard test pattern using a size III white stimulus was performed. HVFs were included for analysis if they were considered reliable as defined by less than 15% false-positive rates and 30% fixation losses and false-negative rates according to previous criteria.^20^

### Acquisition of Data From the Visual Fields

In this analysis, the raw values of the patient's retinal sensitivity at each of the HVF 24-2 predetermined points were extracted from pdf scans of the HVFs using a custom data extraction tool based on the Python^21^ package “hvf extraction script.”^22^ This script used Google's tesseract optical character recognition^23^ to distinguish text inside a digital image and return the relevant text in a useable format. Although the “hvf extraction script” was not originally intended for use on scanned documents, cleaning the images before processing gave values for the majority of the visual field locations. A manual validation of the total cohort point retinal sensitivity eliminated missing data and discrepancies between the original values and the data extraction tool.

### HVF Analysis

To detect pointwise change over the course of the study, the points were categorized by individual pointwise retinal sensitivity at baseline. The mean change in sensitivity was plotted at each point from baseline to 12 and 24 months. Subsequently, the cohort was restricted to a population defined by a baseline MD between −2 dB and −7 dB to simulate a medically managed population. Finally, the number of points in the visual field in the whole cohort and in the restricted simulated medically treated cohort that would be expected to change per sensitivity category were calculated.

### Statistical Analysis

Analysis of clinical data was based on the full dataset according to the statistical analysis plan.^7^ In this evaluation, analyses were based on a per-protocol analysis. Statistical analysis was performed using R 3.6.3 (R Foundation for Statistical Computing, Vienna, Austria). Data were reported with mean and SD for normally distributed variables and median and range for data that were not normally distributed. Missing clinical data, due to any absence or choice, were excluded from the analysis.

## Results

**Figure 1.**
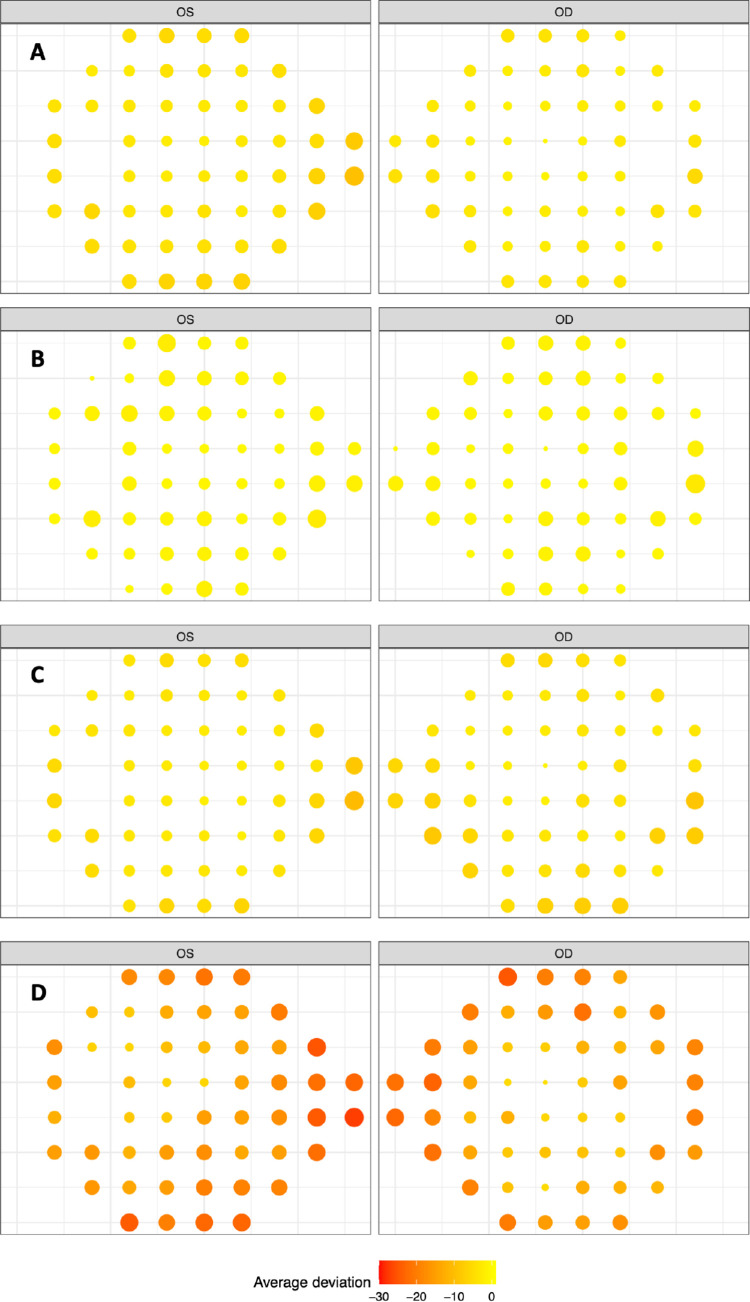
**(A)** The retinal sensitivity at baseline for each location of the HVF for the whole cohort, within eye. All patients were used. **(B)** The retinal sensitivity at baseline for each location of the visual field for those with MDs of −2 dB or better. **(C)** The retinal sensitivity at baseline for each location of the visual field for those with MDs between −2 dB and −7 dB. **(D)** The retinal sensitivity at baseline for each location of the visual field for those with MDs worse than −7 dB.

Characteristics of the study population are summarized in Table 1. Retinal sensitivity values for the whole cohort at baseline showed that the central points were less affected than the peripheral points (Fig. 1A). The whole cohort was then categorized according to the extent of their reduced visual function at baseline as per MD category (Figs. 1B–1D). As the visual function declined, the distribution of the average deviation points became increasing prominent in the periphery and around the blind spot.

**Table 1.** IIH:WT Baseline Characteristics

Characteristic	Total (*N* = 66)
Age at baseline (y), mean ± SD	32 ± 7.8
Duration of IIH diagnosis (y), median (IQR)	1.1 (0.5–2.6)
Number on acetazolamide (%)	19 (29)
Opening lumbar puncture pressure (cm CSF), mean ± SD	35.5 ± 7.0
Weight (kg), mean ± SD	118.5 ± 21.1
BMI, mean ± SD	43.9 ± 7.0
Perimetric MD worse eye (dB),	−3.6 ± 3.7
mean ± SD	(*n* = 65)^a^
Frisén grade, worse eye, mean ± SD	2.1 ± 1.0

IQR, interquartile range; CSF, cerebrospinal fluid; BMI, body mass index.

a Missing data are indicated in parentheses.

### Pointwise Location Sensitivity for Whole Cohort at 12 and 24 Months

**Figure 2.**
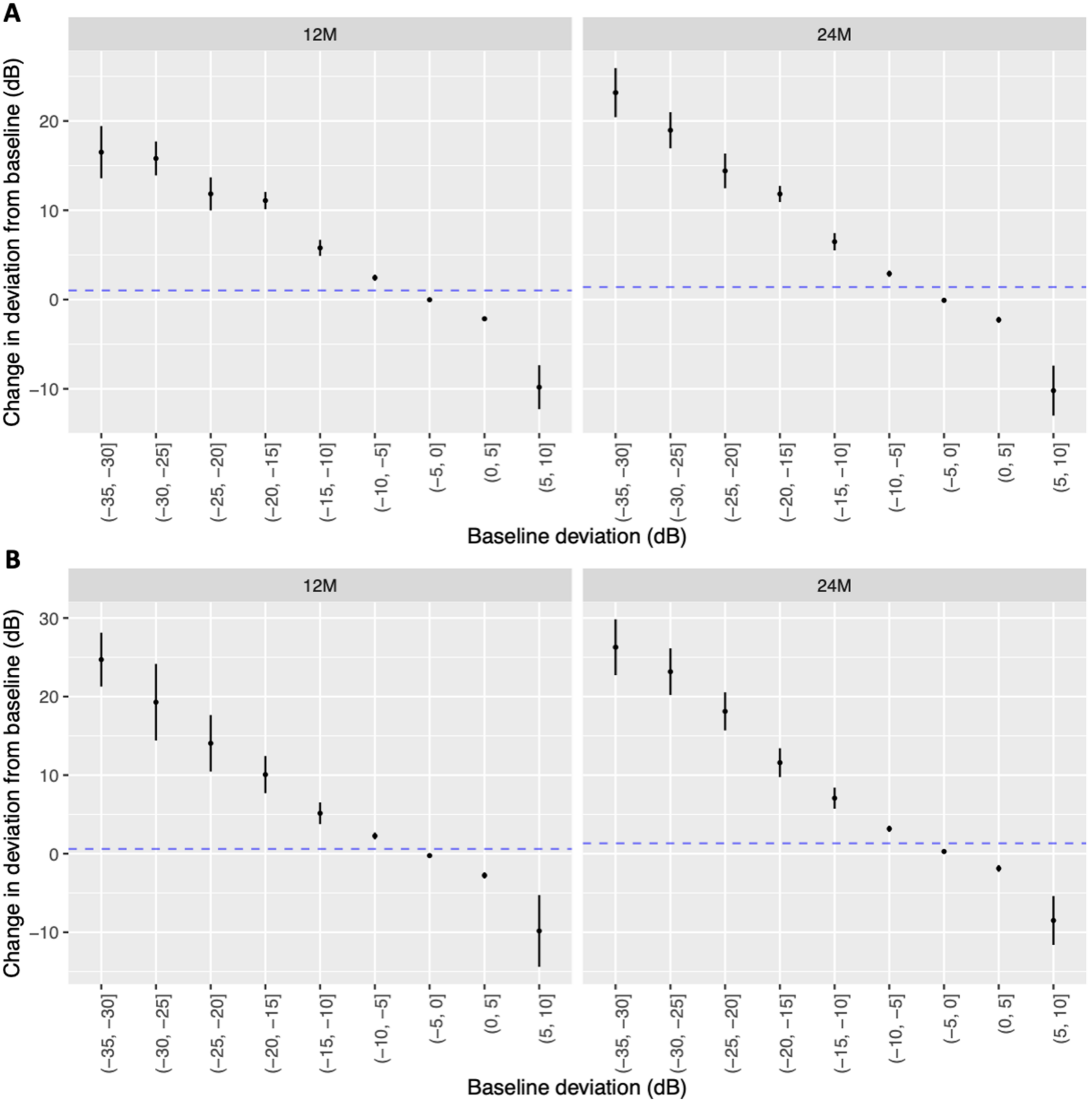
**(A)** The mean change in deviation from baseline (and 95% confidence intervals) to 12 months and 24 months in subsets of points classified by baseline deviation. All patient eyes were used. Categories with at least 10 observations at each time point are shown. The sizes of the groups are naturally different, and this is reflected in the widths of the confidence intervals. **(B)** The mean change in deviation from baseline (and 95% confidence intervals) to 12 months and 24 months in the population defined by MDs between −2 dB and −7 dB at baseline (simulation of a medically treated population).

Points with baseline sensitivities between 0 dB and −10 dB showed small changes over time points (0–5 dB, mean 0.02 ± 3.1; −5 to −10 dB, mean 2.4 ± 4.7 at 12 months) (Fig. 2, Table 2). Points with sensitivities worse than −10 dB demonstrated a larger improvement over time; for example, at 12 months, between −10 dB and −15 dB, the mean ± SD was 5.78 ± 6.10 dB, and from −15 to −20 dB, the mean was 11.10 ± 5.02 dB (Fig. 2, Table 2). For points with baseline sensitivities of −35 to −30 dB, there was a large SD (mean, 16.51 ± 13.75 dB) (Fig. 2, Table 2) at 12 months. When points with baseline sensitivity between −10 dB and −25 dB were analyzed for the whole cohort, the mean change at 12 months was 8.53 ± 6.75 dB, increasing further to 9.61 ± 6.99 dB by 24 months (Tables 2, 3).

**Table 2.** Number of Points and Mean Change in Point Sensitivity in Visual Field Test Locations Categorized by the Baseline Point Sensitivity Subgroup at 12 and 24 Months

Time (mo)	Baseline Point Sensitivity Subgroup (dB)	Number of Points	Mean at Baseline (dB)	Mean Change From Baseline	SD
12	(−35, −30)	85	−32.2	16.5	13.7
	(−30, −25)	56	−26.8	15.8	7.2
	(−25, −20)	67	−21.9	11.8	7.7
	(−20, −15)	103	−17.0	11.1	5.0
	(−15, −10)	176	−11.7	5.8	6.1
	(−10, −5)	711	−6.4	2.4	4.7
	(−5, 0)	3412	−1.7	−0.0	3.0
	(0, 5)	661	1.6	−2.2	3.1
	(5, 10)	11	7.5	−9.8	4.2
24	(−35, −30)	63	−32.0	23.2	11.1
	(−30, −25)	53	−26.8	19.0	7.5
	(−25, −20)	61	−21.9	14.4	7.7
	(−20, −15)	102	−17.0	118	4.6
	(−15, −10)	166	−11.7	6.5	6.4
	(−10, −5)	652	−6.4	2.9	4.5
	(−5, 0)	2784	−1.7	−0.1	3.8
	(0, 5)	480	1.7	−2.3	3.7
	(5, 10)	10	7.4	−10.2	4.5

**Table 3.** Number of Points and Mean Change in Point Sensitivity Over Time in Test Locations With a Baseline Point Sensitivity Between −10 dB and −25 dB, Categorized by Trial Arm and Use of Acetazolamide

Time (mo)	Group	Number of Points Between −10 dB and −25 dB	Mean	SD
12	Whole cohort	346	8.5	6.8
	CWI	127	8.2	7.2
	CWI with acetazolamide	22	8.8	4.3
	CWI with no acetazolamide	105	8.0	7.6
	Bariatric surgery	219	8.8	6.5
24	Whole cohort	329	9.6	7.0
	CWI	118	11.5	7.1
	CWI with acetazolamide	15	7.0	4.5
	CWI with no acetazolamide	103	12.2	7.2
	Bariatric surgery	211	8.6	6.7

#### Analysis of Pointwise Sensitivities in the Simulated Medically Managed Cohort

Those with a MD between −2 dB and −7 dB at baseline had a similar distribution of changes in the point-sensitive deviation at baseline (Fig. 2B). Overall, the vast majority of data points that were included were in the 0 to −10 dB category (*n* = 4123), compared with points between −10 dB and −25 dB (*n* = 346) and those between −25 dB and −5 dB (*n* = 487) (Table 4).

**Table 4.** Subanalysis (Defined by MD Between −2 dB and −7 dB at Baseline) to Simulate a Medically Treated Cohort Where the Number of Point Sensitivities Are Categorized by the Location Point Sensitivity

Baseline Point Sensitivity Subgroup (dB)	Time Point (mo)	Number of Points That Could Be Analyzed	Mean (dB)	SD
0 to −10	12	4123	0.4	3.5
0 to −10	24	3436	0.5	4.1
−10 to −25	12	346	8.5	6.8
−10 to −25	24	329	9.6	7.0
−25 to −35	12	487	10.8	9.1
−25 to −35	24	445	12.6	9.3

#### Analysis of Pointwise Sensitivities in the Whole Cohort

The utility of baseline points between −0 dB and −10 dB was examined to establish how point-sensitivity analysis performed in IIH:WT. As expected, these demonstrated very little change at 12 and 24 months (at 12 months, the mean change was 0.4 ± 3.5 dB; at 24 months, the mean change was 0.48 ± 4.11 dB). Baseline sensitivities between −10 dB and −25 dB have the ability to change over time (Fig. 2). It was only when using the whole cohort that the largest mean changes were found: 8.53 ± 6.75 dB at 12 months and 9.60 ± 6.99 dB at 24 months (Table 3). However, there were fewer points available for analysis (*n* = 346 at 12 months and *n* = 329 at 24 months) compared with cases where the baseline pointwise sensitivity ranged from 0 to −10 dB (*n* = 4123 at 12 months and *n* = 1844 at 24 months) (Table 4). Furthermore, there was little difference observed between trial arms when analyzing the points that were between −10 dB and −25 dB at baseline, as the bariatric surgery arm was 8.75 ± 6.51 dB at 12 months and the CWI group was 8.16 ± 7.15 dB at 12 months. This was despite a significant difference between baseline and 12 months in the ICP of −6.0 cm cerebrospinal fluid (CSF) between the trial arms. Among those in the CWI arm who were not on acetazolamide, the point sensitivity mean change between baseline and 12 months was 8.03 ± 7.26 dB. Despite the significant reduction in ICP found between the bariatric surgery group and the CWI group, there was little discrimination analyzing point sensitivities among bariatric surgery, CWI, CWI with no acetazolamide treatment, and CWI with concurrent use of acetazolamide (Table 5).

**Table 5.** Longitudinal Mean Pointwise Location Sensitivity Changes in Those With Point Sensitivities Between −10 dB and −25 dB, Categorized by Treatment at 12 and 24 Months

	Total Cohort		Bariatric Surgery		All CWI		CWI and No Concurrent Acetazolamide Treatment		CWI and Concurrent Use of Acetazolamide	
	Mean ± SD (*n*)	Effect Size	Mean ± SD (*n*)	Effect Size	Mean ± SD (*n*)	Effect Size	Mean ± SD (*n*)	Effect Size	Mean ± SD (*n*)	Effect Size
12 mo	8.53 ± 6.8 (346)	1.27	8.75 ± 6.5 (219)	1.34	8.16 ± 7.2 (127)	1.14	8.77 ± 4.3 (22)	2.04	8.03 ± 7.6 (105)	1.05
24 mo	9.60 ± 6.99 (329)	1.37	8.55 ± 6.7 (211)	1.27	11.5 ± 7.1 (118)	1.62	12.16 ± 7.2 (103)	1.69	7.00 ± 4.52 (15)	1.55

### Categorizing the Population by Baseline MD

To understand how representative a baseline point sensitivity beyond −10 dB in one or more points was in an active IIH population, we calculated the number of points ≥ −10 dB in each individual (Table 6). In the whole cohort, the median number of points on the baseline visual field worse than −10 dB was 5 (interquartile range, 2–13), with 57% of the cohort having at least two points worse than −10 dB at baseline (Table 6). In the subgroup with a MD between −2 dB and −7 dB at baseline, 42% had more than five points that were worse than −10 dB. As the number of points required for analysis decreased, more participants were available for inclusion; for example, 73% had at least two or more points, and 85% had one point worse than −10 dB (Table 6). Also, 31% of patients at 12 months and 38% at 24 months would have achieved five or more points that improved by 7 dB (Table 7).

**Table 6.** Number of Participants Who Had One or More Baseline Points in Either Eye With a Sensitivity Worse Than −10 dB in the Entire Cohort With and Without Perimetric MD Criteria

		Number of Points ≤ −10 dB in Either Eye at Baseline (%)					
Population	*n*	1	2	3	4	5	Median Points (IQR)^a^
Whole cohort	58	39 (0.67)	33 (0.57)	26 (0.45)	20 (0.35)	20 (0.35)	5 (2–13.5)
MD ≥ −2 dB	38	33 (0.87)	29 (0.76)	22 (0.58)	19 (0.50)	19 (0.50)	6 (2–15)
MD between 2 dB and 7 dB	33	28 (0.85)	24 (0.73)	17 (0.52)	14 (0.42)	14 (0.42)	4 (2–10)
MD ≤ −7 dB	6	6 (1.00)	6 (1.00)	6 (1.00)	6 (1.00)	6 (1.00)	50.5 (32.75–71.25)

a The median number of points ≤ −10 dB in either eye at baseline (and IQR) in only those patients who had at least one qualifying point.

**Table 7.** Percentage and Number of Participants Who Had Pointwise Improvement of 7 dB or More From Baseline at 12 and 24 Months

	Patients Who Had a Pointwise Improvement of 7 dB or More From Baseline at	
Number of Available Points on the HVF 24-2 at Baseline	12 mo (*N* = 53), % (*n*)	24 mo (*N* = 44), % (*n*)
0	26 (14)	18 (8)
1	13 (7)	9 (4)
2	6 (3)	7 (3)
3	4 (2)	7 (3)
4	1 (7)	1 (4)
5	1 (4)	1 (5)
>5	30 (16)	37 (17)

## Discussion

In this study, we characterized the pointwise pattern of visual field change in a cohort of people with active IIH recruited to the IIH:WT. Those with baseline point sensitivities between 0 dB and −10 dB showed small changes over time and, as expected, were unlikely to demonstrate clinically meaningful change over both 12 and 24 months. Points in the −10 to −25 dB category demonstrated change that could be considered clinically meaningful (mean of 8.5 dB in at least one point in the whole visual field); however, using data between −10 dB and −25 dB resulted in fewer data points and larger SDs for analysis. Although the median number of points worse than −10 dB was five, 43% of all of the IIH:WT participants had fewer than two points worse than −10 dB at baseline, emphasizing that data points worse than −10 dB were not representative of the majority of IIH patients.

It should be emphasized that eligibility for the IIH:WT was not determined by MD criteria. Therefore, to simulate the HVF data to reflect a typically medically managed cohort, we chose a baseline HVF in which the MD was between −2 dB and −7 dB (the criterion range used in the IIHTT^9^). Among this group, 42% had five or more points worse than −10 dB at baseline (Table 7). If only two points were required for analysis, 73% had two or more points worse than −10 dB in either eye at baseline (Table 6). Thus, we found that it would be challenging to use point analysis as an outcome for an interventional medical trial in IIH, as the pool of point-sensitivity data available for meaningful analysis would be extremely small. Additionally, the participants overall would be less representative of the whole disease spectrum, which could affect the applicability of the results being directly translatable to clinical practice. Finally, test locations with 8 to 18 dB of loss at baseline had a 95% prediction interval that nearly covered the full measurement range of the instrument (0–40 dB)^24^; thus, the test–retest variability of these locations was so poor that there was little signal above the variability-related noise.^25^

There is no universally adopted, minimally clinically important change in HVF measures in IIH as there are in glaucoma.^17^^,^^26^^,^^27^ In glaucoma, visual field progression equal to or faster than −0.5 dB per year for at least five abnormal test locations at baseline has been found to be clinically significant,^28^ as have changes from baseline beyond the 5% probability levels for the Glaucoma Change Probability analysis in five or more reproducible visual field locations.^29^ Although pointwise analysis in patients with IIH has revealed changes around the blind spot and in the nasal area, likely reflecting the reduction in optic head nerve swelling as the papilledema resolves,^10^ visual fields with global diffuse damage, such as occur in patients with IIH, tend to be more variable than fields with focal damage such in glaucoma.^30^ The fundamental differences between these diseases confound the applicability of glaucoma outcome measures to IIH trials. IIH is a rare condition compared with glaucoma which immediately affects the trial design and recruitment potential, particularly as other tools that assess visual function, such as visual acuity (Snellen or logMAR), color vision, and contrast sensitivity, have not been found to be discriminatory in medically managed IIH.^9^^,^^30^

A limitation of this study is that it included only patients with well-established IIH. Thus, the results may not be applicable to patients with recently diagnosed IIH or to severely affected patients who may require urgent surgery.^6^^,^^31^ In addition, because our cohort was small, it was subject to regression to the mean with respect to the mean deviation (Fig. 2). Regression to the mean is a common statistical phenomenon that occurs when repeated measurements are made on the same subject. Subjects would not be expected to have the same measurements at two different times due to measurement error and random fluctuation. Regression to the mean needs to be considered to distinguish a real change from the expected change due to the natural variation in test readings. To minimize regression to the mean, participants should be randomized to study arms, with a control arm being fundamental to the design of the trial. Variability can be further reduced by selecting participants using two or more baseline measurements, resulting in better estimates of the mean and the within-subject variation.

In this study and in studies reported by others,^10^ the visual field deficit in IIH typically occurs across the full VF and increases with eccentricity.^13^ Unfortunately, these are the very points that show the largest variability in visual field testing.^32^^,^^33^ Visual field tests also have been found to be unreliable when visual field locations have sensitivity below 15 to 19 dB because of a reduction in the asymptotic maximum response probability.^34^ In addition to test limitations, there are demonstrable changes in cognition in the domains of attention and executive function that have been found in patients with IIH and that directly affect the performance indicators in HVF testing.^35^

Our data indicate that point analysis of the HVF has no advantage over global MD analysis, at least in the population we studied from the IIH:WT. As expected, baseline points that were better than −10 dB had little room to improve over time and, thus, offered little utility for analysis. If the generic threshold for a clinically meaningful change of 7 dB is recommended for IIH treatment trials, baseline points in the range of −10 to −25 dB would be needed for analysis. The US Food and Drug Administration has stated that visual field loss has likely occurred if ≥5 visual field locations have significant change beyond the 5% probability level or if there is at least a 7-dB between-group mean difference for the entire field.^17^ A pointwise approach for people with IIH is not feasible as demonstrated here because there are too few data points available for analysis. Additionally, the points that could be used are known to be more variable. In our study, even when a limited threshold was set to determine a clinically meaningful change, point analysis did not offer an advantage over global MD. Consequently, point sensitivity analysis in medically treated IIH is likely to be prohibitive in clinical trials and not representative of the IIH disease spectrum. In addition, if the requirement of regulators for a meaningful change in MD is a 7-dB difference between trial arms, as recommended for glaucoma treatment trials,^17^ then using MD as a primary outcome would not be achievable in medical IIH trials, as these typically recruit participants with MDs between −2 dB and −7 dB. Future studies may consider investigating the use of a larger stimulus size that has been demonstrated to retain the ability to detect defects, lower retest variability, and improve the useful dynamic range of the instrument.^36^^,^^37^
